# Object-Place Recognition Learning Triggers Rapid Induction of Plasticity-Related Immediate Early Genes and Synaptic Proteins in the Rat Dentate Gyrus

**DOI:** 10.1155/2008/269097

**Published:** 2009-01-26

**Authors:** Jonathan Soulé, Zsuzsa Penke, Tambudzai Kanhema, Maria Nordheim Alme, Serge Laroche, Clive R. Bramham

**Affiliations:** ^1^Department of Biomedicine and Bergen Mental Health Research Center, University of Bergen, Jonas Lies vei 91, 5009 Bergen, Norway; ^2^CNRS, Université Paris-Sud, UMR 8620, Laboratoire de Neurobiologie de l’Apprentissage, de la Mémoire et de la Communication, 91405 Orsay Cedex, France

## Abstract

Long-term recognition memory requires protein synthesis, but little is known about the coordinate regulation of specific genes. Here, we examined expression of the plasticity-associated immediate early genes (Arc, Zif268, and Narp) in the dentate gyrus following long-term object-place recognition learning in rats. RT-PCR analysis from dentate gyrus tissue collected shortly after training did not reveal learning-specific changes in Arc mRNA expression. In situ hybridization and immunohistochemistry were therefore used to assess possible sparse effects on gene expression. Learning about objects increased the density of granule cells expressing Arc, and to a lesser extent Narp, specifically in the dorsal blade of the dentate gyrus, while Zif268 expression was elevated across both blades. Thus, object-place recognition triggers rapid, blade-specific upregulation of plasticity-associated immediate early genes. Furthermore, Western blot analysis of dentate gyrus homogenates demonstrated concomitant upregulation of three postsynaptic density proteins (Arc, PSD-95, and *α*-CaMKII) with key roles in long-term synaptic plasticity and long-term memory.

## 1. INTRODUCTION

Memory consolidation is thought to rely on long-lasting, activity-dependent modifications of synaptic strength and remodeling of neural network connectivity. For example, both hippocampal-dependent learning and long-term potentiation (LTP) are associated with cytoarchitectural reorganization of synapses, including thickening of the postsynaptic density and expansion of the dendritic spine head. Such stable structural alterations typically require new gene expression, protein synthesis, as well as local actin polymerization [1–5]. Several lines of evidence implicate rapid, activity-dependent expression of immediate early genes (IEGs) in consolidation of memory and long-term synaptic plasticity.

IEGs encode a diverse set of gene products that include secreted proteins, cytoplasmic enzymes, and inducible transcription factors. Critical roles in consolidation of memory and LTP have been identified for two IEGs, activity-regulated cytoskeleton associated protein/activity-regulated gene 3.1 (Arc/Arg3.1), and Zif268 (also known as Egr1, Krox24, and NGFI-A). Thus, gene knockout or knockdown (antisense) of Arc [6, 7] or Zif268 [8, 9] produces selective defects in diverse types of long-term memory as well as in maintenance of late phase LTP in the dentate gyrus (DG). Upon induction, Arc mRNA is rapidly transported to dendrites where it undergoes local translation [10–12]. The Arc protein is implicated in control of actin polymerization at synapses and regulation of AMPA-type glutamate receptor trafficking [13–16]. Zif268, a zinc-finger transcription factor of the Egr family, is implicated in the control of gene networks [17, 18]. Arc and Zif268 are now widely used as markers of neuronal activation and plasticity during memory formation [19–21].

The neurotrophin, brain-derived neurotrophic factor (BDNF), is a major regulator of protein synthesis-dependent consolidation of hippocampal memory [22–25]. For example, a BDNF-dependent de novo protein-synthesis phase is necessary for memory formation, consolidation, and persistence of hippocampus-dependent inhibitory avoidance learning [26–29]. Recent work has revealed a stringent requirement for Arc synthesis in LTP elicited by either BDNF infusion or high-frequency stimulation (HFS) in the dentate gyrus [13]. Another IEG induced by BDNF infusion into the dentate gyrus is neuronal activity-regulated pentraxin (Narp) [30]. Narp has been implicated in synapse formation and maturation during development and induces clustering of AMPA receptors at excitatory synapses [31–33]. Interestingly, however, the immediate early gene Zif268, which plays a critical role in HFS-induced LTP and long-term memory, is not upregulated in response to in vivo infusion of BDNF [30, 34].

Recognition memory can be assessed in rodents in various behavioral tasks such as novel object recognition [35] or object-place recognition, tasks based on rats' innate propensity to explore novel rather than familiar objects or to preferentially explore displaced objects. The involvement of the hippocampal formation in the neural circuitry supporting recognition memory has been shown by lesion studies [36–40]. In terms of molecular mechanisms, certain molecules such as the MAPK/ERK [41], the transcription factor CREB [42, 43] as well as the IEGs Arc [7], Zif268 [9, 44], and Egr3 [45] have been shown to be crucial for the formation of long-term object recognition memory.

Studies on the molecular mechanisms of recognition memory have relied mainly on the behavioral analysis of knockout mice. Thus, little is known about the coordinate regulation and dynamics of gene expression and protein synthesis. Here, we studied the coordinate expression of Arc, Narp, and Zif268 in the dentate gyrus after training rats in an object-place recognition task. In this task leading to the formation of long-term object-place recognition memory, rats explored three different objects in a familiar environment. Animals remember the nature of the encountered objects as well as their location in the environment, thus placing a demand on spatial memory and hippocampal function [44, 46].

A key feature of dendritic remodeling occurring during learning is likely to be the coordinate synthesis and integration of protein constituents of the postsynaptic density (PSD) complex of excitatory synapses. Arc localizes to the PSD and is thought to play a key role in LTP by stabilizing nascent filamentous actin [3, 13, 47–49]. In addition to Arc, the PSD proteins PSD-95 and *α*-CaMKII play major roles in regulating the composition and function of the postsynaptic element during LTP and memory formation [50–53]. All of these proteins can also be synthesized from local mRNAs in dendrites [11, 12, 54–56]. We therefore investigated coordinate regulation of key protein constituents of the PSD, Arc, *α*-CaMKII, and PSD-95, in the dentate gyrus following recognition learning.

## 2. EXPERIMENTAL PROCEDURES

### 2.1. Animals

Adult male Sprague-Dawley rats (*n* = 80; Iffa-Credo, France) weighing 300–350 g at the beginning of the experiment (mean age 8 weeks, range 7.5–9 weeks) were used as subjects. After arrival in the laboratory, they were housed in pairs under constant temperature and lighting conditions (22°C, light/dark cycle of 12:12 hours, lights on at 07:00). Rat chow and tap water were provided *ad libitum*. All efforts were made to minimize the number of animals and their suffering throughout the experiments. Experiments were performed in accordance with the European Communities Council Directive of November the 24th 1986 (86/609/EEC) and the French National Committee (87/848). All experiments were conducted during the light phase.

#### 2.1.1. Long-term memory for spatial configuration of objects

To test long-term object-place recognition memory, we used a modified version of the standard object recognition task [35], based on the discrimination between a novel and a familiar spatial location of an object [44]. Fifteen rats were handled twice daily for 4 days, followed by a 3-day rest, before the beginning of the experiments. The experimental apparatus was a cylindrical open field made of metal and painted black (diameter 90 cm, height 40 cm), with wood shavings on the floor, and located in a room with dim lighting and constant background noise. A cue card was placed at a fixed location on the top of the wall of the open field to facilitate spatial mapping of each object. Rats were habituated to the open field in the absence of objects for 2 × 5-minute exploration a day for 3 days. The next day (acquisition session), three objects were placed in the open field, and rats were allowed to explore them for four 5-minute sessions with 5-minute intervals. The objects consisted of assembled interlocking plastic block pieces (Lego-blocks) of different shapes and colors. Retention testing, lasting 5 minutes, was conducted 2 or 3 days after the acquisition session in the same arena with the spatial position of one object changed to a new position. Care was taken to displace objects in a counterbalanced manner across animals, so that each of the objects was displaced in a randomized manner in terms of nature of the object and position to avoid any bias that could arise if some animals would have shown a preference for an object or a place. To examine retention performance at the two delays in the same animals, rats were tested twice in the acquisition-retention sequence with different sets of objects. There was a 2-day rest interval between the retention session and the next acquisition session of the first sequence and the acquisition session of the second sequence. During the acquisition and retention phase, the time spent exploring each object was recorded. The criteria for exploration were based strictly on active exploration, where rats had both forelimbs within a circle of 5 cm around an object, head oriented toward it or touching it with their noses.

Time spent exploring each object was expressed in percent of total time spent exploring all the objects. Exploration time for each object during the acquisition session was analyzed using ANOVA. For the retention session, exploration of the displaced object was expressed as a percentage of the total time of object exploration and compared with chance level (33.33%) using Student's one-sample *t*-test. The significance level was set at *P* < .05.

#### 2.1.2. Experimental groups for in situ hybridization and immunohistochemistry

Rats were submitted to one of five different treatments. Cage control rats (CC, *n* = 4) were handled daily as described in the methods (on the same days as the other animals), and were taken directly from their home cage and sacrificed on the same days as rats from the other groups. Trained rats were submitted to habituation and the object recognition acquisition session as described above, and sacrificed 10 minutes (L10, *n* = 4) or 60 minutes (L60, *n* = 4) after the end of the acquisition session. Control rats matched to the trained rats were handled and habituated as described above, and on the day following the last habituation session, they were re-exposed to the same open field without objects, which they explored according to the same time schedule as L10 and L60 rats (four 5-minute sessions with 5-minute intervals) and were killed 10 minutes (C10, *n* = 4) or 60 minutes (C60, *n* = 4) later.

Rats were perfused transcardially under urethane anesthesia (1 mg/kg body weight) with 0.1 M phosphate buffer (PB; pH 7.4) containing 1 mM orthovanadate, then with phosphate buffer containing 4% paraformaldehyde. Brains were postfixed in the same fixative solution overnight at 4°C, transferred to a phosphate buffer containing 0.1% sodium azide, and stored at 4°C. Brains were incubated in PB containing 30% sucrose overnight at room temperature. On the following day, coronal sections (30 *μ*m-thick) were obtained on a Leica CM3050S cryostat equipped with a Richard-Allan Sec35e blade. Chamber and object temperatures were set to −20°C and −14°C, respectively. Sections were immediately stored in PB containing 0.1% sodium azide at 4°C. For immunohistochemistry and in situ hybridization, sections corresponding to the dorsal hippocampus (between approximately −3.3 mm and −4.5 mm from Bregma) were selected.

#### 2.1.3. Experimental groups for RT-PCR and western blotting

Rats were submitted to the same behavioral protocols as for in situ hybridization and immunohistochemistry (*n* = 9 for each group). Animals were decapitated under urethane anesthesia; their brain was quickly removed and rinsed with ice-cold, sterile 0.9% saline. The hippocampus was quickly removed and the dentate gyrus was dissected on ice, frozen in liquid nitrogen and stored at −80°C.

#### 2.1.4. Poly(A) RNA and cDNA preparation

Poly(A) RNA was isolated using the Dynabeads mRNA direct kit (Dynal, Oslo, Norway) according to the manufacturer's protocol with minor modifications. 70 *μ*l magnetic beads were used per sample and the isolated poly(A) RNA fraction was eluted in 2 × 30 *μ*l of 10 mM Tris/HCl, pH 8.0. The yield and quality of the poly(A) RNA were determined by measuring the absorbance at 260/280 nm. 60 ng poly(A) RNA was reversed-transcribed using the Superscript First-Strand Synthesis Kit (Invitrogen) and the resulting cDNA was diluted 20-fold.

#### 2.1.5. Semiquantitative real-time PCR and normalization strategies

Semiquantitative real-time PCR was performed on an iCycler (Bio-Rad) using cDNA from individual animals and the iQ SYBR Green Supermix. 5 *μ*l cDNA were added to the PCR reaction mix to yield a total of 25 *μ*l. PCR quantification was performed in triplicate, and the fluorescence signal was quantified by the second derivative maximum method using the iCycler iQ Real-Time detection system software. Primers used are given in Table 1. Data were normalized with the geometric mean of the three normalization genes polyubiquitin, Cyclophilin, and HPRT. Primer sequences in 5′ to 3′ direction and annealing temperatures are also given in Table 1.

### 2.2. Riboprobes

Arc riboprobes were prepared from a cDNA insert matching the first 2975 nucleotides of the Arc mRNA (GenBank accession number NM_019361) cloned into the pCRII-TOPO vector (Invitrogen). Antisense and sense probes were transcribed from linearized plasmids using T7 and SP6 polymerases in the presence of DIG labelling mix according to the manufacturer's instructions (Roche).

### 2.3. In situ hybridization

Floating sections were placed in PBS for 5 minutes, treated with proteinase K (10 *μ*g/mL) for 5 minutes at 37°C, and postfixed (5 minutes with 4% PFA/PBS). After postfixation, sections were treated with 0.25% acetic anhydride in 0.1 M TEA (pH = 8.0) for 10 minutes, washed twice in 2xSSC, and placed for 10 minutes in prehybridization buffer. Riboprobes were applied onto the sections and hybridization was performed in a humidified chamber at 60°C for at least 16 hours. Sections were washed twice with 2xSSC at RT for 30 minutes, once with 50% formamide in 2xSSC at 65°C, rinsed in 2xSSC at 37°C, incubated with 20 *μ*g/mL RNase A at 37°C for 30 minutes and incubated in RNase A buffer for at 65°C for 30 minutes. After blocking in 2% blocking reagent for one hour at RT, AP-coupled anti-DIG antibody (1:2000, Roche) was applied. Visualization was accomplished with the chromogenic substrates NBT and BCIP (Roche). Control performed with the Arc sense riboprobes did not provide any staining. Arc-positive cells exhibited characteristic staining in the soma, the perinuclear region and/or in nuclear foci.

### 2.4. Antibodies

Primary antibodies used for immunoblotting were as follows: Arc H-300 (sc-15325, 1:200, Santa Cruz), *β*-actin (clone AC-15, 1:5000, Sigma), PSD-95 (MA1-045, 1:500, Affinity BioReagents) and *α*-CaMKII (mouse monoclonal, IgG1 MA1-048, 1:2000). For immunochemistry, the antibodies were as follows: Zif268 (sc-110, 1:200, Santa Cruz) and Narp (polyclonal antibody, 1:250, gift from Richard O’Brien, Johns Hopkins University).

### 2.5. Immunohistochemistry

Sections were first treated with PB containing 100 mM glycine (Sigma), then washed in PBT (PB containing 0.1% Tween 20), incubated in 0,3% H2O2 diluted in PBT, permeabilized for 20 minutes with 0.5% Triton X-100 diluted in PBT, rinsed and immersed for 30 minutes in blocking buffer (4% BSA and 4% donkey serum in PBT). They were then incubated overnight at 4°C with the primary antibody diluted in blocking buffer. After three washes in PBT, biotinylated secondary antibody was applied for 1 hour at RT. Sections were then washed in PBT, incubated for 1 hour in Streptavidin-HRP diluted in PBT, washed in PBT and finally processed for DAB staining. Zif268-positive cells were defined by their characteristic nuclear staining whereas Narp-positive cells presented somatic staining.

#### 2.5.1. Image acquisition and analysis

Pictures were taken on a Nikon Eclipse 80i microscope coupled to a Nikon DS-5M camera. Representative pictures were acquired with a 4× objective whereas evaluation of the density of granule cells positively marked by in situ hybridization and immunohistochemistry was carried out using 10× and 20× objectives. The NIS-elements Ar2.3 software (Nikon) was used for determination of positively stained cells and the area covered by the granule cell layer. Counting of stained cells was accomplished by systematic scanning of the entire thickness of nonconsecutive sections to avoid under- and overestimation of the cell densities. The density calculation was based on the number of positive cell bodies or nuclei within the area bounded by the granule cell layer of the upper or lower blade of the dentate gyrus.

#### 2.5.2. SDS-PAGE and western blotting

Protein levels in homogenate samples were determined using the BCA Protein Assay kit (Pierce). Equal amounts of protein were loaded onto SDS-PAGE gels (10%) and run overnight at constant 10 mA. Separated proteins were transferred to a nitrocellulose membrane (Hybond-C, Amersham-GE Healthcare, Oslo, Norway) at a constant voltage of 20 V overnight or 100 V for one hour. Membranes were blocked on a gyro-rocker for 1 hour at room temperature (RT). Blocking buffer (BB) consisted of TBST (Tris-buffered saline/0.1% Tween-20) and 5% BSA or 5% nonfat dry milk. The primary antibodies were dissolved in BB containing 5% BSA and the blots incubated for 2 hours at RT or 4°C overnight with constant shaking. Following three washes with TBST, blots were incubated for 1 hour in horseradish peroxidase-conjugated secondary antibody dissolved in TBST. The blots were washed three times with TBST and proteins were visualized using enhanced chemiluminescence (ECL Western Blotting Analysis System, Pierce ECL Western Blotting Substrate). Blots were stripped with Restore Plus Western Blot Stripping buffer (Pierce, Rockford, USA) at room temperature for 20 minutes and reprobed with another antibody detecting the protein of interest. Optical density values for each protein were normalized relative to values obtained with *β*-actin antibody.

### 2.6. Statistical analysis

All data are presented as mean ± SEM. Statistical analysis was based on ANOVA and Tukey's post hoc test was used for further comparisons between the C10, L10, C60, and L60 groups. The CC group used for normalization was independently compared with the other groups using ANOVA. The significance level was set at *P* ≤ .05.

## 3. RESULTS

### 3.1. Long-term memory for the spatial configuration of objects

**Figure 1:**
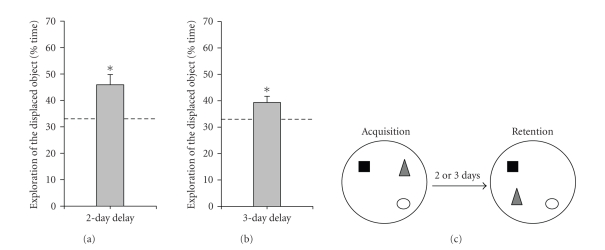
Performance at the 2-day and 3-day retention intervals of the object-place recognition memory task. At both (a) 2-day and (b) 3-day delays after acquisition, rats (*n* = 15 in each case) showed preferential exploration of the displaced object. (c) Schematic representation of the task. Asterisks indicate *P* ≤ .05 compared with chance level (dashed line, 33.3%). Data are presented as mean ± SEM.

The training procedure involved habituation to the test arena, followed by exposure to three objects at fixed locations on four consecutive 5-minute sessions with 5-minute intervals (acquisition phase), and a retention test, which was performed 2 or 3 days later. In the retention test, one of the objects was displaced and the amount of time exploring the displaced object relative to the total time of object exploration was determined. This paradigm has been previously shown to induce long-term memory for objects and location of objects [44]. During the acquisition phase, ANOVA did not show significant differences between the time spent exploring the three objects (time spent exploring the three objects for the 2-day delay: 33.0 ± 2.0%, 38.9 ± 2.2%, 28.1 ± 1.3% of total time; *F*_2,42_ = 0.90, *P* = .41, for the 3-day delay: 32.7 ± 1.5%, 21.1 ± 1.2%, 46.2 ± 1.4% of total time; *F*_2,42_ = 2.68, *P* = .08). During retention testing, rats spent significantly more time exploring the displaced object than chance level (33.33%) at both the 2 and 3-day retention intervals (time spent exploring the displaced object for the 2-day delay: 45.9 ± 1.3% of total time; *t*_14_ = 3.27, *P* < .01; for the 3-day delay: 39.3 ± 2.3% of total time; *t*_14_ = 2.54, *P* < .05), as shown in Figures 1(a) and 1(b). This behavioral analysis shows that rats in our experimental conditions were able to form a long-term object-place recognition memory.

### 3.2. Object recognition training induces Arc mRNA expression in granule cells of the dorsal blade of the DG

**Figure 2:**
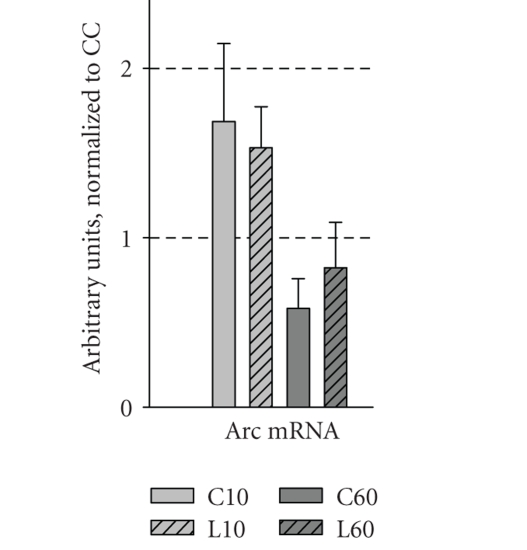
Expression levels of Arc in the dentate gyrus after object recognition. Fold change in mRNA levels (relative to the CC group) is presented for Arc in the dentate gyrus of animals from all five groups (*n* = 8 for all groups, except L60, *n* = 7). Data are presented as mean ± SEM. Gene expression was normalized to control genes (see methods).

We hypothesized that acquisition of different types of information about the objects and their spatial location would be associated with rapid induction of the immediate early gene Arc. This issue was first addressed by semiquantitative RT-PCR analysis of Arc mRNA levels in the microdissected DG. Surprisingly, no significant change in Arc mRNA levels could be observed in C10, C60, L10, and L60 animals when compared with caged control (CC) animals (Figure 2, *P* > .05), indicating that Arc expression in the dentate gyrus was not significantly affected by exploration of the arena with or without the objects.

**Figure 3:**
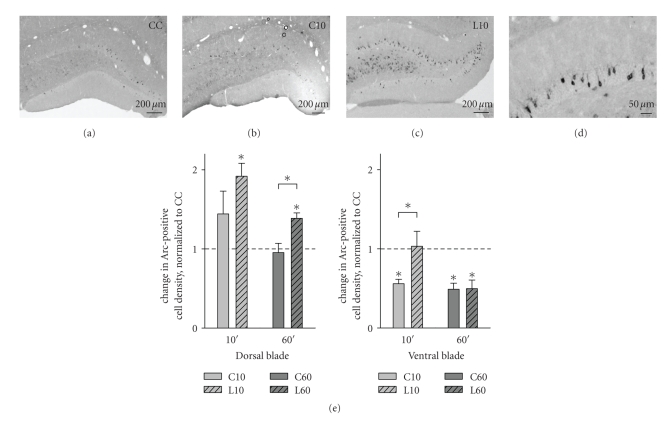
Object recognition training increases Arc mRNA expression in the dentate gyrus. Arc mRNA in situ hybridization reveals sparse expression of Arc in both dorsal and ventral blades of the rat dentate gyrus of (a) CC, (b) C10, and (c) L10 animals. (d) Higher magnification shows typical Arc mRNA localization in the cell body and dendrites of granule cells. (e) Change in Arc-positive granule cell densities (relative to the CC group) in the dorsal and the ventral blade of the dentate gyrus across the C10, L10, C60, and L60 groups. Data are presented as mean ± SEM (*n* = 4 for all groups). Asterisks indicate *P* ≤ .05 (if not indicated otherwise, relative to CC group). Scale bars represent 200 *μ*m in (a)–(c) and 50 *μ*m in (d).

Endogenous Arc-expressing granule cells represent a very low percentage (1–2%) of the total number of granule cells in the DG. Following spatial behavioral experience, the density of Arc-expressing cells increases specifically in the dorsal (inner) blade, while the density in ventral (outer) blade remains nearly unchanged [57]. We considered that such sparse, blade-specific changes in gene expression may not be detected by PCR analysis of whole DG homogenate samples. We therefore re-examined the effect of learning about objects and their configuration on Arc mRNA expression using in situ hybridization (Figure 3). As previously described, Arc-expressing cells were dispersed along both the dorsal and the ventral blades of the DG of both the CC and trained groups (Figures 3(a)–3(d)). The granule cell layer of CC animals presented an average density of 152.2 ± 10.5 Arc-positive cells per mm^2^ in the dorsal blade whereas the ventral blade presented an average density of 138.8 ± 13.8 Arc-positive cells per mm^2^. Figure 3(e) shows the normalized density of Arc mRNA-positive granule cells in the dorsal and ventral blades of the DG following performance of the recognition task. ANOVA revealed a blade effect (*F*_(1,31)_ = 64.310; *P* < .001), a time effect (*F*_(1,31)_ = 14.181; *P* < .001) and a learning effect (*F*_(1,31)_ = 10.417; *P* = .004). In the dorsal blade, a significant 2-fold increase in density was detected in L10 animals, relative to the CC group (*P* < .01), while the C10 group exhibited a nonsignificant 1.4-fold increase relative to CC. The density of Arc mRNA-positive cells in the dorsal blade remained elevated up to one hour after training in the learning group. L60 animals displayed a significant 1.4-fold increase when compared with CC levels (*P* < .01) and a 1.5-fold increase in comparison to C60 levels (*P* = .02). In the ventral blade, a surprising decrease in the density of Arc-positive cells was observed in the C10 and C60 group, relative to caged controls (*P* < .01), indicating that the exploration of the environment induced a rapid and sustained decrease in Arc expression that was specific to the ventral blade. Nonetheless, exposure to the objects resulted in a 2-fold increase in Arc-expression at 10 minutes (*P* = .05) relative to rats exposed only to the test arena. Interestingly, no effect of the presence of the three objects was observed in the ventral blade at the 60-minute time point. Thus, learning about objects in this recognition task resulted in rapid and sparse increase in Arc mRNA expression in both blades of the DG. However, only the dorsal blade of the DG exhibited a sustained increase in Arc mRNA expression up to one-hour posttraining.

### 3.3. Object recognition training increases Zif268 protein expression in granule cells of the dorsal and ventral blades of the DG

**Figure 4:**
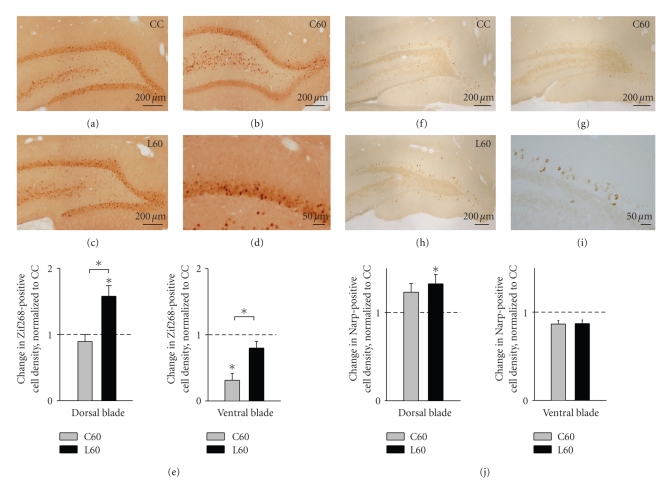
Object recognition training induces Zif268 and Narp protein expression in the dentate gyrus. Zif268 immunohistochemistry reveals the presence of Zif268 protein in granule cells in both blades of the dentate gyrus of (a) CC, (b) C60, and (c) L60 animals. (d) Higher magnification shows the presence of Zif268 in the nucleus of granule cells. (e) Change in density of Zif268-positive nuclei (relative to the CC group) in the dorsal and the ventral blade of the dentate gyrus across the C60 and L60 groups. Narp immunohistochemistry reveals the presence of Narp protein in granule cells in both blades of the dentate gyrus of (f) CC, (g) C60, and (h) L60 animals. (i) Higher magnification shows the presence of Narp in the cell body of granule cells. (j) Change in density of Narp-positive cells (relative to the CC group) in the dorsal and the ventral blade of the dentate gyrus across the C60 and L60 groups. Data are presented as mean ± SEM (*n* = 4 for all groups). Asterisks indicate *P* ≤ .05 (if not indicated otherwise, relative to CC group). Scale bars represent 200 *μ*m in (a)–(c) and (f)-(h) and 50 *μ*m in (d) and (i).

Zif268 protein expression in the DG was monitored by immunohistochemistry (Figure 4, left panel). Zif268 protein showed typical nuclear localization in the granule cells of both blades of the DG (Figures 4(a)–4(d)). In caged control animals, the dorsal blade presented an average density of 295.9 ± 42,8 positive cells per mm^2^ whereas the ventral blade presented an average density of 336.3 ± 68 positive cells per mm^2^. Comparison of C60 and L60 animals by ANOVA showed a learning effect (*F*_(1,15)_ = 24.183; *P* < .001) as well as a blade effect (*F*_(1,15)_ = 23, 061; *P* < .001). In the dorsal blade, a significant 1.8-fold increase in the density of Zif268-positive granule cells was observed in the L60 group (*P* = .01) when compared with the expression in the C60 group which was equivalent to that of the caged controls (Figure 4(e)). In the ventral blade, a similar recognition learning-specific increase was seen when L60 animals were compared with the C60 controls (*P* = .01). However, as also observed for Arc, the density of Zif268-expressing cells in the ventral blade was significantly reduced in the C60 group relative to caged controls (*P* = .02, Figure 4(e)). These results show that object recognition induces Zif268 expression in both blades of the DG.

### 3.4. Object recognition training increases Narp protein expression in granule cells of the dorsal blade of DG

Narp staining was obvious in cells of both blades of the DG and was restricted to the cell bodies (Figures 4(f)–4(i)). Caged control animals exhibited an average density of 582.5 ± 52.9 Narp-positive cells per mm^2^ in the dorsal blade and 510.9 ± 93.26 positive cells per mm^2^ in the ventral blade. A modest increase of Narp-positive granule cells was detected in the dorsal blade of C60 and L60 groups, relative to caged controls (Figure 4(j)). The increase observed in the group of animals exposed to the objects (L60) was significant (*P* = .03), whereas expression in the C60 group was not significantly different from caged controls. In contrast to Arc and Zif268, no changes in Narp expression were detected in the ventral blade in the C60 and L60 groups. However, ANOVA did not detect any learning-specific change indicating that the task had an effect on Narp protein expression specific to the dorsal blade of the DG, which cannot be attributed solely to acquisition of the object-place configuration.

### 3.5. Object recognition training increases levels of Arc, *α*-CaMKII, and PSD-95 protein expression in the DG

**Figure 5:**
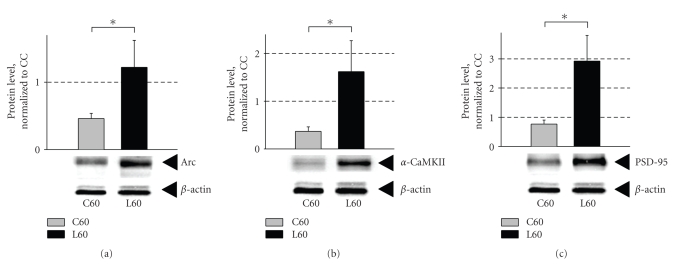
Object recognition training induces an increase in the expression of Arc, *α*-CaMKII, and PSD-95 proteins in the dentate gyrus. Representative blots and comparison of normalized protein levels of (a) Arc, (b) *α*-CaMKII, and (c) PSD-95 proteins are presented for the C60 and L60 groups (relative to CC). Data are presented as mean ± SEM (*n* = 7 for all groups). Protein levels were normalized to *β*-actin. Asterisks indicate *P* ≤ .05.

Western blot was used to assess the expression levels of Arc protein in the DG of trained animals (Figure 5(a)). Arc levels were elevated more than 2.5-fold in the L60 group compared with C60 (*P* = .05). This learning-associated increase matches the changes in Arc mRNA as revealed by in situ hybridization (Figure 3(d)). We then asked whether this increase in Arc expression is paralleled by altered expression of other proteins involved in synaptic plasticity and memory consolidation. For this purpose we chose two core constituents of the postsynaptic density complex, the scaffolding protein PSD-95 and the enzyme *α*-CaMKII. Both proteins undergo local dendritic synthesis, regulate the structure and receptor composition of the PSD, and have important functions in synaptic plasticity and memory [50–53, 58–61]. Like Arc, *α*-CaMKII (Figure 5(b)) and PSD-95 (Figure 5(c)) were both upregulated in the L60 group relative to the C60 group, which was exposed to the arena without objects (*P* = .03 and *P* = .02). Another intriguing aspect of the protein response was the decrease in expression of Arc and *α*-CaMKII in the C60 group to as much as 50% of the caged controls, although this effect was not significant.

## 4. DISCUSSION

The main findings of the present study are as follows. (1) Object recognition training induces sparse IEG expression in the granule cell layer of the DG as shown histochemically by the upregulation of Arc, Zif268, and to a lesser extent, Narp. (2) Object exploration induces Zif268 expression across both blades of the dentate gyrus, whereas Arc and Narp expression are selectively induced in the dorsal blade. (3) The levels of Arc, *α*-CaMKII, and PSD-95, three synaptically located proteins that are crucial for long-term memory are concomitantly increased in DG homogenates one hour after object recognition training.

### 4.1. Object recognition training enhances immediate early gene expression in the DG

RT-PCR did not show significant up- or downregulation of Arc mRNA (Figure 2). While this negative result suggested that granule cells are unresponsive, RT-PCR may fail to detect changes that are restricted to subpopulations of granule cells, or possible bidirectional changes within the population. Our in situ hybridization and immunohistochemistry approach revealed that Arc, Zif268, and Narp are all upregulated in dentate granule cells shortly after completion of the object recognition task (Figures 3 and 4). Furthermore, the distinct spatial patterns of activation testify to a strong differential control of IEG expression across the dorsal and ventral blade of the DG. Arc mRNA was only transiently increased in the ventral blade, but showed sustained expression in the dorsal blade. Narp protein showed the same dorsal blade-specific pattern, whereas Zif268 was elevated equally in both DG blades.

Chawla et al. [57] have previously demonstrated sparse expression of Arc in the dorsal, but not ventral, blade of the DG following a spatial behavioral experience in a novel environment. In that study, rats exploring two different arenas exhibited environment-specific increase of Arc expression in the dorsal blade. Thus, enhancement of Arc expression in the dorsal blade of the DG is common to spatial exploration of a novel environment as well as object-place recognition learning. Interestingly, no significant increase of Arc expression was observed in our C10 and C60 group after exploration of a known environment, which suggests that Arc induction in the DG is specific to novel spatial experience. As discussed in the paper of Chawla et al., blade-specific alterations in gene expression might be related to differences in the density of excitatory synapses onto granule cells or differences in local circuitry between the blades. Additionally, Fevurly and Spencer reported that stress also has an opposite effect on Fos expression in the two blades of the dentate gyrus [62]. Previous work has shown that Arc and Narp, but not Zif268, are strongly upregulated during BDNF-LTP [30, 34, 63]. Further work is needed to determine if effects of learning about new objects on Arc and Narp expression reflect selective activation of endogenous BDNF signaling in the dorsal blade of the dentate gyrus.

Interestingly, Fos immunostaining in the DG is higher in rats presented with familiar, but not novel arrangements of familiar items [64, 65]. This work involved the display of items on remote pictures whereas rats in our study were free to explore the objects in an unchanged configuration. Nevertheless, our results showing increases in Arc and Zif268 expression in the DG after object-place recognition are in line with the proposal that the DG is involved in the discrimination of the relative familiarity of spatial arrangements [65]. By showing the regulated expression of several genes and proteins, the present results confirm the responsiveness of the DG in the context of object-place recognition memory.

### 4.2. Rapid expression of synaptic proteins in the DG after object recognition training

Dendritic spines are subject to activity-driven synaptic reorganization and growth through mechanisms involving BDNF signaling, local protein synthesis, and actin polymerization. We have observed parallel regulation of Arc, *α*-CaMKII, and PSD-95 in the DG following recognition learning (Figure 5). These proteins are all constituents of the PSD, they can be synthesized from dendritic mRNA, and each of them has important functions in long-term modification of synaptic structure and efficacy [47–49, 51, 53, 66]. Recent evidence suggests that conversion of short-term to long-term memory requires a protein synthesis phase in a limited posttraining time window in the hippocampus and that persistence of memory is BDNF-dependent [27, 28]. BDNF-induced LTP in the DG requires Arc synthesis, which serves to stabilize the newly polymerized actin [13]. Arc and *α*-CaMKII are also both locally translated in response to BDNF application to synaptoneurosomes [55, 67, 68]. Our data therefore support the model that recognition memory involves rapid and coordinate regulation of plasticity-related PSD proteins.

Besides the object learning-specific increases in protein expression, there was a trend toward decreased gene and protein expression in animals exposed to the empty arena. The mechanisms underlying these decreases are unknown at present. There appears to be a blade-specific component to this as the density of Arc- and Zif268-expressing granule cells was significantly decreased only in the ventral blade. Arc and *α*-CaMKII protein expression were similarly reduced to below 50% in DG homogenates obtained from rats repeatedly exposed to the empty arena. This is interesting given recent evidence that memory formation and LTP maintenance require proteasomal degradation of proteins [69–72], especially in the context of memory reactivation, which presumably occurred in our control rats that were repeatedly exposed to the arena [69–72]. The current view of synaptic modification combines highly regulated protein synthesis with specific proteasomal degradation. It is therefore conceivable that degradation of Arc and *α*-CaMKII following repeated exposure to the empty arena plays some role in preparing synapses for subsequent protein synthesis-dependent remodeling. Alternatively, it has been previously demonstrated that prolonged exposure of animals to an open-field results in decreased levels of phosphorylated CREB, which may act to decrease CREB responsive genes [73]. There is recent evidence for the presence of a CRE site in the Arc promoter. Thus, downregulation of Arc expression could be the result of CREB hypophosphorylation in control animals [74].

In conclusion, we have provided evidence that the granule cells of the DG are responsive to learning about, and forming a long-term memory of objects and that the formation of this type of memory triggers upregulation of the synaptic-plasticity related IEGs Arc and Zif268 along with enhanced expression of the synaptic proteins PSD-95 and *α*-CaMKII. Interestingly, in some cases upregulation associated with object-place learning appeared to be superimposed on downregulation of expression induced by the known context. Further work is needed to define the precise behavioral roles of gene and protein regulation in the object-recognition paradigm. Pollak and colleagues [75] recently reported coordinate expression of BDNF, Zif268, PSD-95, and pCaMKII in the hippocampus after spatial training in the Morris water maze. The similarities to the present study of object-place recognition memory give support to the notion that similar molecular mechanisms underlie diverse forms of hippocampus-dependent long-term memory.

## Figures and Tables

**Table 1:** Overview over primer sequences and accession numbers for the analyzed genes.

Gene	Primer sequence	Ann temp. (C°)	Acc. number
Arc	Fw: CCCAGTCTGTGGCTTTTGTCA	60	NM019361
	Bw: GTGTCAGCCCCAGCTCAATC		
Cyclophilin	Fw: AGCACTGGGGAGAAAGGATT	60	BC059141
	Bw: GATGCCAGGACCTGTATGCT		
Polyubiquitin	Fw: GGCAAGACCATCACCCTAGA	60	BC070919
	Bw: GCAGGGTTGACTCTTTCTGG		
HPRT	Fw: GCAGACTTTGCTTTCCTTGG	60	NM_012583
	Bw: TCCACTTTCGCTGATGACAC		

## ABBREVIATIONS

BDNF: Brain-derived neurotrophic factor
RT-PCR: Real-time polymerase chain reaction
LTP: Long-term potentiation
IEG: Immediate early gene
HFS: High-frequency stimulation
AMPA: *α*-amino-3-hydroxy-5-methyl-4-isoxazolepropionate
PSD: Postsynaptic density
